# Teriparatide ameliorates articular cartilage degradation and aberrant subchondral bone remodeling in DMM mice

**DOI:** 10.1016/j.jot.2022.10.015

**Published:** 2022-12-07

**Authors:** Guoqing Li, Su Liu, Yixiao Chen, Huihui Xu, Tiantian Qi, Ao Xiong, Deli Wang, Fei Yu, Jian Weng, Hui Zeng

**Affiliations:** a Department of Bone & Joint Surgery, Peking University Shenzhen Hospital, Shenzhen, People's Republic of China, 518036; b National & Local Joint Engineering Research Center of Orthopaedic Biomaterials, Peking University Shenzhen Hospital, Shenzhen, People's Republic of China, 518036

**Keywords:** Teriparatide (PTH (1–34)), Knee osteoarthritis, Cartilage, Subchondral bone, Osteoprotegerin (OPG), **KOA**, knee osteoarthritis, **OP**, osteoporosis, **SCB**, subchondral bone, **PTH (1–34)**, Teriparatide, **WT**, wild type, **OPG**^**−/−**^, osteoprotegerin-knockout, **DMM**, destabilization of medical meniscus, **GAG**, glycosaminoglycan, **ECM**, extracellular matrix, **OPG**, osteoprotegerin, **ARRIVE**, Animal Research: Reporting of *In Vivo* Experiments, **PCR**, polymerase chain reaction, **S.I**, subcutaneous injection, **HPLC**, High Performance Liquid Chromatography, **ROI**, region of interest, **Micro-CT**, microcomputer tomography, **BMD**, bone mineral density, **Tb.N**, trabecular number, **Tb.Th**, trabecular thickness, **BV/TV**, bone volume fraction, **SMI**, structure model index, **HE**, hematoxylin and eosin, **EDTA**, ethylene diamine tetra acetic acid, **TB**, toluidine blue O, **SOFG**, Safranin O-fast green, **AB**, Alican blue, **MT**, masson's trichrome, **OARSI**, Osteoarthritis Research Society International, **nM**, nMol/L, **PBS**, phosphate buffer solution, **DMEM**, Dulbecco's Modiﬁed Eagle's Medium, **FBS**, fatal bovine serum, **EdU**, 5-ethynyl-2′-deoxyuridine, **CLSM**, confocal laser scanning microscope, **GAPDH**, glyceraldehyde-3-phosphate dehydrogenase, **IL-1β**, Interleukin-1β, **CCK-8**, cell counting kit-8, **OD**, optical density, **IL-6**, Interleukin-6, **TNF-α**, tumor necrosis factor-α, **ELISA**, enzyme-linked immunosorbent assay, **RT-qPCR**, quantitative reverse transcription polymerase chain reaction, **NCBI**, National Center for Biotechnology Information, **AGC**, Aggrecan, **COLII**, Type II collagen, **AGC**, aggrecan, **SOX9**, SRY-Box Transcription Factor 9, **MMP13**, Matrix Metallopeptidase 13, **COLX**, Type X collagen, **ADAMTS5**, ADAM Metallopeptidase with Thrombospondin Type 1 Motif 5, **ANOVA**, one-way analysis of variance

## Abstract

**Objective:**

Knee osteoarthritis (KOA) is a highly prevalent musculoskeletal disorder characterized by degeneration of cartilage and abnormal remodeling of subchondral bone (SCB). Teriparatide (PTH (1–34)) is an effective anabolic drug for osteoporosis (OP) and regulates osteoprotegerin (OPG)/receptor activator of nuclear factor ligand (RANKL)/RANK signaling, which also has a therapeutic effect on KOA by ameliorating cartilage degradation and inhibiting aberrant remodeling of SCB. However, the mechanisms of PTH (1–34) in treating KOA are still uncertain and remain to be explored. Therefore, we compared the effect of PTH (1–34) on the post-traumatic KOA mouse model to explore the potential therapeutic effect and mechanisms.

**Methods:**

*In vivo* study, eight-week-old male mice including wild-type (WT) (*n* ​= ​54) and OPG^−/−^ (*n* ​= ​54) were investigated and compared. Post-traumatic KOA model was created by destabilization of medial meniscus (DMM). WT mice were randomly assigned into three groups: the sham group (WT-sham; *n* ​= ​18), the DMM group (WT-DMM; *n* ​= ​18), and the PTH (1–34)-treated group (WT-DMM ​+ ​PTH (1–34); *n* ​= ​18). Similarly, the OPG^−/−^ mice were randomly allocated into three groups as well. The designed mice were executed at the 4th, 8th, and 12th weeks to evaluate KOA progression. To further explore the chondro-protective of PTH (1–34)*,* the ATDC5 chondrocytes were stimulated with different concentrations of PTH (1–34) *in vitro*.

**Results:**

Compared with the WT-sham mice, significant wear of cartilage in terms of reduced cartilage thickness and glycosaminoglycan (GAG) loss was detected in the WT-DMM mice. PTH (1–34) exhibited cartilage-protective by alleviating wear, retaining the thickness and GAG contents. Moreover, the deterioration of the SCB was alleviated and the expression of PTH1R/OPG/RANKL/RANK were found to increase after PTH (1–34) treatment. Among the OPG^−/−^ mice, the cartilage of the DMM mice displayed typical KOA change with higher OARSI score and thinner cartilage. The damage of the cartilage was alleviated but the abnormal remodeling of SCB didn't show any response to the PTH (1–34) treatment. Compared with the WT-DMM mice, the OPG^−/−^-DMM mice caught more aggressive KOA with thinner cartilage, sever cartilage damage, and more abnormal remodeling of SCB. Moreover, both the damaged cartilage from the WT-DMM mice and the OPG^−/−^-DMM mice were alleviated but only the deterioration of SCB in WT-DMM mice was alleviated after the administration of PTH (1–34). *In vitro* study, PTH (1–34) could promote the viability of chondrocytes, enhance the synthesis of extracellular matrix (ECM) (AGC, COLII, and SOX9) at the mRNA and protein level, but inhibit the secretion of inflammatory cytokines (TNF-α and IL-6).

**Conclusion:**

Both wear of the cartilage was alleviated and aberrant remodeling of the SCB was inhibited in the WT mice, but only the cartilage-protective effect was observed in the OPG^−/−^ mice. PTH (1–34) exhibited chondro-protective effect by decelerating cartilage degeneration *in vivo* as well as by promoting the proliferation and enhancing ECM synthesis of chondrocytes *in vitro.* The current investigation implied that the rescue of the disturbed SCB is dependent on the regulation of OPG while the chondro-protective effect is independent of modulation of OPG, which provides proof for the treatment of KOA.

**The translational potential of this article:**

Systemic administration of PTH (1-34) could exert a therapeutic effect on both cartilage and SCB in different mechanisms to alleviate KOA progression, which might be a novel therapy for KOA.

## 1 Introduction

Knee osteoarthritis (KOA) is a highly prevalent musculoskeletal disorder, which has impaired activities and quality of life of the patient and is ranked one of the most common orthopedic disorders [1]. It is a whole-joint disorder with cartilage erosion and subchondral bone (SCB) disturbance, the pathogenesis and treatment of which is affected by multi-factors such as senility, gender, genetics, obesity, and trauma, etc [2]. Cartilage is highly-organized connective tissue and chondrocytes are the unique cellular component to maintain the balance of the extracellular matrix (ECM) [3], and the SCB plays a crucial role in KOA progression [4,5]. Synovial cells can secret the related cytokines by inducing the expression of receptor activator of nuclear factor ligand (RANKL) to promote osteoclast formation and bone resorption [6]. The integrity and physiology of cartilage and SCB is important for the maintenance of the joint, which is a challenging aspect in KOA treatment. Lesions of these structures would result in KOA initiation with clinical symptoms while the treatment of KOA is challenged with less desirable effects. Current strategies such as exercise and analgesic drugs are common choices with no ideal effect [7]. Therefore, disease-modified medicines for the prevention and treatment of OA are highly desirable.

Teriparatide (PTH (1–34)), the recombinant human parathyroid hormone (1–34), is applied for the treatment of osteoporosis (OP) and bone fracture [8]. Investigations of PTH (1–34) on wild-type (WT) KOA mice have been extensively studied and a multitude of research revealed that PTH (1–34) might play its role in regulating the differentiation of chondrocytes [9,10]. Nevertheless, the mechanism of PTH (1–34) functions in KOA is still uncertain. Osteoprotegerin (OPG) is capable of binding the RANKL to regulate the differentiation of osteoclast [11] and maintain the integrity of articular cartilage [12]. PTH (1–34) is well applied in OP treatment and regulated OPG/RANKL/RANK signaling [13], but whether the mechanisms of PTH (1–34) involved the role of OPG during the KOA treatment is still uncertain and remains to be explored.

Therefore, in this study, both WT and OPG-knockout (OPG^−/−^) mice models were used to evaluate the potential therapeutic distinction of PTH (1–34) including cartilage-protective, chondro-regenerative, and SCB amelioration. We hypothesized that PTH (1–34) might attenuate the KOA progression by exerting chondro-protective and SCB-alleviated function, which mechanisms may correlate with the OPG signaling.

## 2 Materials and methods

### 2.1 Animals

All experimental animals were approved by the Animal Care and Use Committee of Peking University Shenzhen Hospital (No. 2021–501) and followed Animal Research: Reporting of *in vivo* Experiments (ARRIVE) guidelines [14]. Eight-week-old male mice including the WT mice (n ​= ​54) and the OPG^−/−^ mice (n ​= ​54) were purchased from the Shanghai Research Center for Biomodel Organisms (Shanghai, China) and genotyped by polymerase chain reaction (PCR). The WT mice were randomly assigned to three groups: the WT-sham group (n ​= ​18), the WT-DMM group (n ​= ​18), and the WT-DMM ​+ ​PTH (1–34) group (n ​= ​18). Similarly, OPG^−/−^ mice (n ​= ​54) were randomly assigned to three groups as well. Mice in the sham group and the destabilization of medial meniscus (DMM) group were administered with an equal volume of saline. All mice were housed in Topbio-technology (Shenzhen, China) under standard laboratory conditions (24 ​°C, 12 ​h light/dark cycle) with food and water. Moreover, none of the mice caught death or infection during the experiment.

### 2.2 DMM surgery and treatment protocol

The mice were anesthetized with isoflurane (RWD life science, Shenzhen, China) and performed with DMM according to the previous protocol, which was widely accepted to establish post-traumatic KOA [15]. *To relieve the pain and remove the sutures before starting the drug treatment, the mice were administrated with anesthesia one week postoper*atively [16,17]. Specifically, all mice were treated by subcutaneous injection (S.I) with Teriparatide (PTH (1–34), HY-P0059, MCE, USA) (100 uL ​× ​40 ​μg/kg/day), which was confirmed by Ultimate 3000 U system High Performance Liquid Chromatography (HPLC) (Thermo Scientific) (Fig. S1). The mice were euthanized in the 4th, 8th, and 12th weeks for relevant evaluation.

### 2.3 Gross observation

All mice were euthanized at the designed time points for the harvest of the knee joints. The tibia plateaus were photographed with a Zoom-stereoscopic microscope (Leica, Germany) and evaluated according to the grading system (Table S1) [18].

### 2.4 Microcomputer tomography evaluation

The region of interest (ROI) was defined between the SCB plate and the epiphysis [19]. The SCB was scanned and three-dimensionally reconstructed by microcomputer tomography (Micro-CT) (vivo CT 80, SCANCO MEDICAL, Switzerland) with a voxel size of 7.8 ​μm, energy/intensity of 70 ​kV, 114 ​μA, and 8 ​W. Finally, parameters of the SCB include bone mineral density (BMD, mg/cm^3^), trabecular number (Tb.N), trabecular thickness (Tb.Th, μm), bone volume fraction (BV/TV, %), and structure model index (SMI) were calculated.

### 2.5 Biosafety evaluation

The primary organs including the heart, liver, spleen, lung, and kidney were obtained. Further, we performed hematoxylin and eosin (HE) staining to evaluate the biosafety of PTH (1–34) *in vivo*.

### 2.6 Histology evaluation

The knee joints were ﬁxed in 10% neutral buffered formalin for one week after the photograph for gross observation, decalcified by 10% ethylene diamine tetra acetic acid (EDTA) (pH ​= ​7.4, 37 ​°C, shaker, 80 ​rpm/min, 3 days), embedded in paraffin, and prepared 3.5 ​μm microsections for staining. The HE staining was applied for a general view while the Toluidine blue O (TB) staining and the Safranin O-Fast Green (SOFG) staining were performed to detect the glycosaminoglycan (GAG) contents of the articular cartilage. Moreover, the Alican blue (AB) staining and Masson trichrome (MT) staining were performed as well. All staining kits were purchased from Solarbio (Beijing, China). Representative changes were photographed by microscope (Leica, Germany) and evaluated by blinded observers according to Osteoarthritis Research Society International (OARSI) system (Table S2) [20]. Cartilage thickness was measured by width-calculating while the special-stained area was measured by an area-calculating algorithm with ImageJ (Version 5.0, USA).

### 2.7 Immunohistochemistry evaluation

Paraffin sections were routinely deparaffinized, rehydrated, and repaired by pancreatic enzyme solution (DIG-3008, MX Biotechnologies, Fuzhou, China) (37 ​°C, 1 ​h). The procedure for IHC evaluation was performed according to the instruction of the Ultra-Sensitive TM SP (Mouse/Rabbit) IHC Kit (KIT-9710, MX Biotechnologies, Fuzhou, China). Moreover, the sections were incubated with Collagen X/COL10A1 Rabbit pAb (A18604) (COLX, 5 ​μg/ml, abcam, USA), Rabbit Anti-MMP13 Polyclonal Antibody (bs-10581 ​R) (MMP13, 10 ​μg/ml, bioss, USA), and Rabbit Anti-ADAMTS5 Polyclonal Antibody (bs-3573 ​R) (COLX, 5 ​μg/ml, bioss, USA) (4 ​°C, overnight, dark). Subsequently, the sections were incubated with PTH/PTHrP-R (L187) pAb (PTH1R, 2.5 ​μg/ml, Bioword Technology, USA), TNFRSF11B Rabbit pAb (A2100) (OPG, 5 ​μg/ml, abcam, USA), RANKL Rabbit pAb (A2550) (RANKL, 5 ​μg/ml, abcam, USA), RANK Rabbit pAb (251971) (RANK, 10 ​μg/ml, Zen Biotechnology, China) (4 ​°C, overnight, dark). Eventually, the brown color was developed with DAB staining (DAB-0031, MX Biotechnologies, Fuzhou, China).

### 2.8 Culture of ATDC5 chondrocytes

The ATDC5 chondrocytes were purchased from Biowing (Shanghai, China) and cultured in Dulbecco's Modiﬁed Eagle's Medium/F12 (DMEM/F12) containing 5% fetal bovine serum (FBS) and 1% penicillin-streptomycin (5% CO_2_, 37 ​°C) (Fig. S2). All materials were purchased from Gibico (Grand Island, USA). PTH (1–34) was diluted into 0.1 ​nM, 0.5 ​nM, 1 ​nM, 3 ​nM, 5 ​nM, and 10 ​nM with DMEM/F12 for further stimulation.

### 2.9 Stimulation of PTH (1–34) on the ATDC5 chondrocytes

#### 2.9.1 Proliferation evaluation

The Live/Dead staining Kit (Invitrogen, USA) was applied to determine the cytotoxicity. The cells were incubated with a staining solution and washed with phosphate buffer solution (PBS) (KeyGen Biotech, China). The 5-ethynyl-2′-deoxyuridine (EdU) Kit (Beyotime Biotechnology, China) was applied to evaluate proliferation. Pictures taken by confocal laser scanning microscope (CLSM) (Leica, Germany) and cells counted by ImageJ (Version 5.0, USA).

#### 2.9.2 ECM synthesis evaluation

The proteins were harvested in radioimmunoprecipitation assay buffer (Beyotime, China) and incubated with the primary antibodies including anti-MMP13 antibody (MMP13, ab84594, 1 ​μg/ml, abcam, UK), anti-ADAMTS5 antibody (ADAMTS5, ab41037, 4 ​μg/ml, abcam, UK), the anti-SOX9 antibody (SOX9, ab185966, 0.5 ​μg/ml, UK), Collagen II Monoclonal Antibody (COLII, MA5-12789, 0.5 ​μg/ml, Invitrogen, USA), TNFRSF11B Rabbit pAb (A2100) (OPG, 1 ​μg/ml, abcam, USA), RANKL Rabbit pAb (A2550) (RANKL, 1 ​μg/ml, abcam, USA), RANK Rabbit pAb (251971) (RANK, 1 ​μg/ml, Zen Biotechnology, China), and glyceraldehyde-3-phosphate dehydrogenase (GAPDH, MA5-15738; Thermo Scientiﬁc, MA, USA) (4 ​°C, shaker, overnight). Bands were incubated with HRP Goat Anti-Mouse IgG (AS003, ABclonal, USA) and detected by the ECL chemiluminescence Kit (EMD Millipore, USA). Finally, the grey scale of the bands measured by ImageJ (Version 5.0, USA).

### 2.10 Treatment of PTH (1–34) on IL-1β-induced-ATDC5 chondrocytes

Chondrocytes were stimulated with Interleukin-1β (10 ​ng/ml) (IL-1β, 211-11 ​B, PeproTech, USA) for 24 ​h to induce the OA chondrocytes [21], followed by 1 ​nM PTH (1–34) stimulation for further 48 ​h.

#### 2.10.1 Viability evaluation

The viability was assessed by cell counting kit-8 assay (CCK-8) (KeyGen Biotech, China) and the optical density (OD) was measured by Microplate Reader (Thermo Scientific Multiskan SkyHigh, USA) at 450 ​nm.

#### 2.10.2 Inflammatory cytokines evaluation

IL-6 kits (EK206/3–01, LIANKE BIO, China) and tumor necrosis factor-α (TNF-α) kits (EK282/4–01, LIANKE BIO, China) were detected by enzyme-linked immunosorbent assay (ELISA). Then, the OD value was read by a Microplate Reader at 450 ​nm, 570 ​nm, and 630 ​nm within 30 ​min.

#### 2.10.3 ECM anabolism evaluation

Quantitative reverse transcription polymerase chain reaction analysis (RT-qPCR) was performed to detect the relative mRNA expression of marked genes. The total mRNA extracted with Trizol (Life Technologies Co., USA) and cDNA was reversed transcript by TaqMan reagents (Takara, Japan). Primers were retrieved from National Center for Biotechnology Information (NCBI) bank and confirmed by the BLAST analysis (Table 1). The relative mRNA expression of Aggrecan (AGC), Type II collagen (COLII), SRY-Box Transcription Factor 9 (SOX9), Matrix Metallopeptidase 13 (MMP13), Type X collagen (COLX), and ADAM Metallopeptidase with Thrombospondin Type 1 Motif 5 (ADAMTS5) were measured. GAPDH was used as endogenous control while the relative mRNA expression (fold control) was calculated with the 2^−ΔΔCt^ method.

**Table 1:** Primers sequence of mark genes.

Gene (ID)	GenBank Accession	NCBI Protein Accession	Coding Length	Sequence		Length
GAPDH (14447)	NM_008085	NP_032111	1317	Forward	AGCTTCGGCACATATTTCATCTG	23
				Reverse	CGTTCACTCCCATGACAAACA	21
MMP13 (17386)	NM_008607	NP_032633	1419	Forward	TGTTTGCAGAGCACTACTTGAA	22
				Reverse	CAGTCACCTCTAAGCCAAAGAAA	23
ADAMTS5 (23794)	NM_011782	NP_035912	2793	Forward	GGAGCGAGGCCATTTACAAC	20
				Reverse	CGTAGACAAGGTAGCCCACTTT	22
COLX (12813)	NM_009925	NP_034055	2043	Forward	TTCTGCTGCTAATGTTCTTGACC	23
				Reverse	GGGATGAAGTATTGTGTCTTGGG	23
COLII (12824)	NM_031163	NP_112440	4464	Forward	GGGAATGTCCTCTGCGATGAC	21
				Reverse	GAAGGGGATCTCGGGGTTG	19
AGC (11595)	NM_007424	NP_031450	6399	Forward	GTGGAGCCGTGTTTCCAAG	19
				Reverse	AGATGCTGTTGACTCGAACCT	21
SOX9 (20682)	NM_011448	NP_035578	1524	Forward	AGTACCCGCATCTGCACAAC	20
				Reverse	ACGAAGGGTCTCTTCTCGCT	20

Notes: NCBI, National Center for Biotechnology Information; GAPDH, glyceraldehyde-3-phosphate dehydrogenase; MMP13, Matrix Metallopeptidase 13; ADAMTS5, ADAM Metallopeptidase with Thrombospondin Type 1 Motif 5; COLX, Type X collagen; COLII, Type II collagen; AGC, Aggrecan; SOX9, SRY-Box Transcription Factor 9.

### 2.11 Statistical analysis

All experiments were performed in triplicates independently. One-way analysis of variance (ANOVA) was performed among multiple comparisons within the WT mice groups or OPG^−/−^ mice groups while two-way ANOVA was applied for multiple comparisons among all the mice groups. Two-tailed *P* ​< ​0.05 were considered statistically significant. Values are labelled as ∗*P* ​< ​0.05, ∗∗*P* ​< ​0.01. Statistical analysis data were measured by R (Version 4.0.2, R Foundation for Statistical Computing, Vienna, Austria) or GraphPad Prism (Version 9.0, GraphPad Software, La Jolla, CA).

## 3 Results

### 3.1 PTH (1–34) alleviates cartilage wear

**Figure 1:**
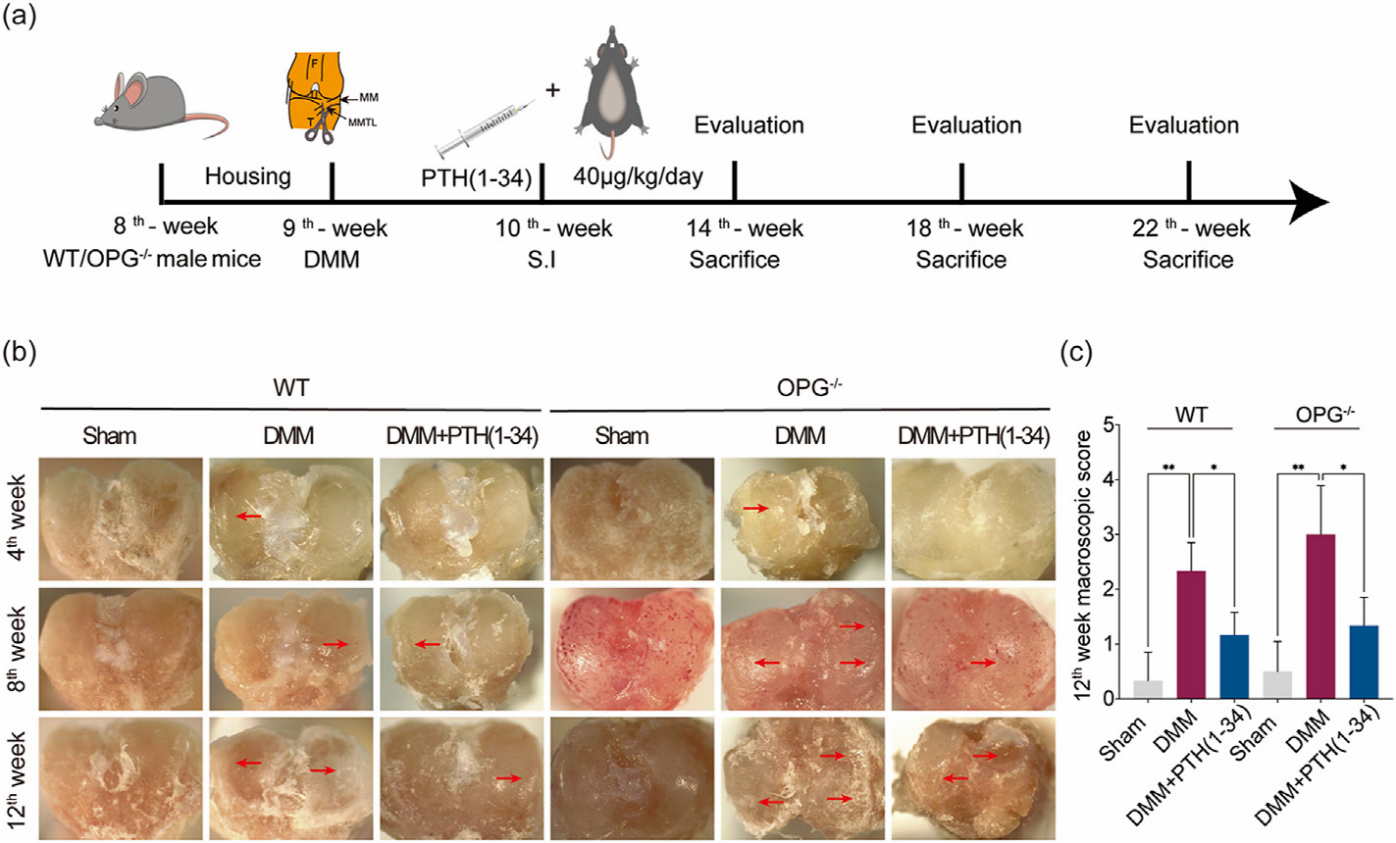
Experimental protocol, gross evaluation, and illustration of tibial plateaus. (a) Schematic of the treatment protocol for mice. (b) and (c) outline of the tibial plateaus and gross evaluation at the 12th week. The surface of the articular cartilage seems much rougher with fibrillation than that in the sham mice but the wear of the cartilage was alleviated in the PTH (1–34) treated mice. Red arrows indicate wear, fibrillation, and roughen of the cartilage. ∗, *P* ​< ​0.05, ∗∗, *P* ​< ​0.01, significant difference compared with sham group. (For interpretation of the references to colour in this figure legend, the reader is referred to the Web version of this article.)

**Figure 2:**
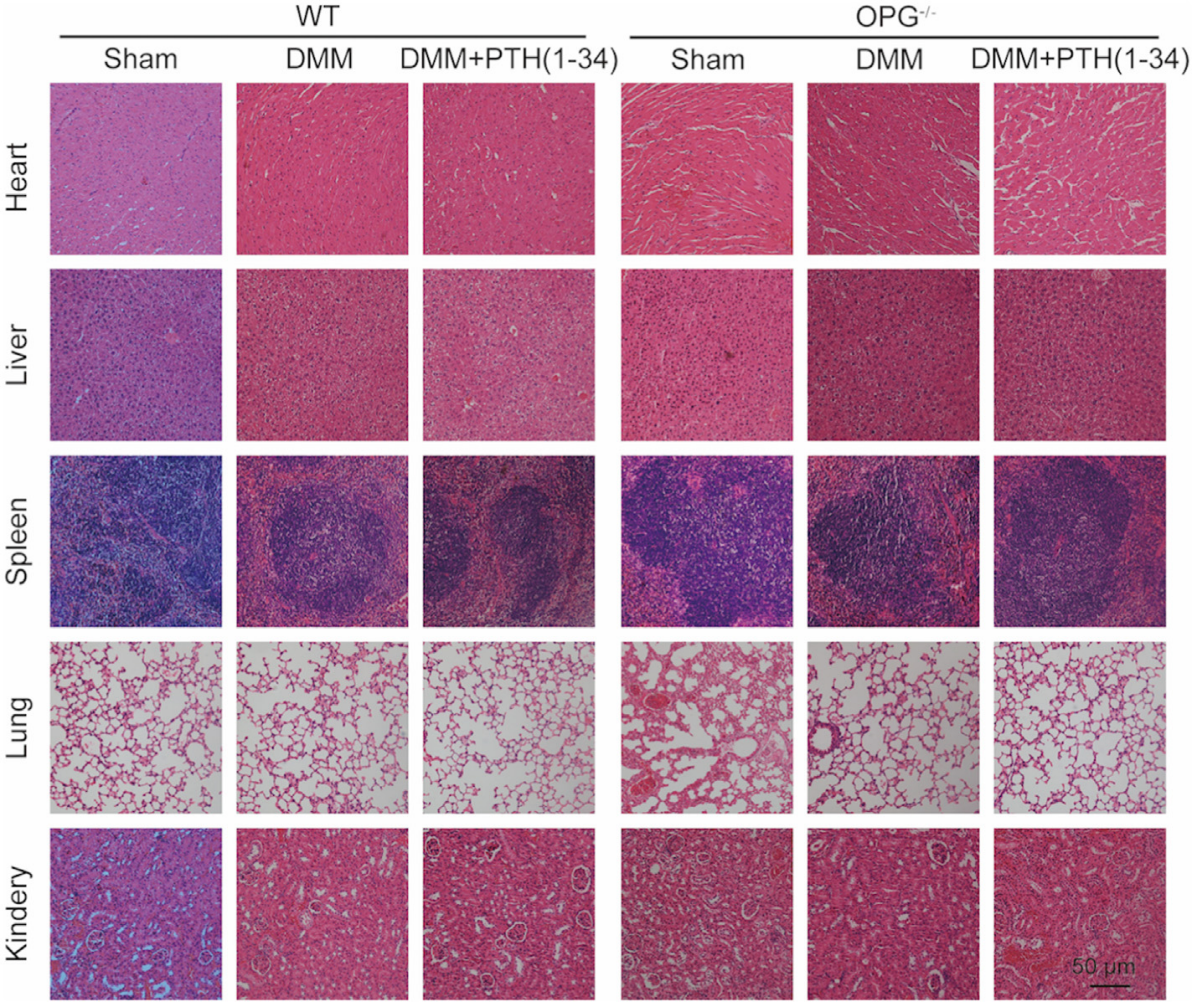
The biosafety evaluation of PTH (1–34) *in vivo*. Representative photographs of pathological changes of heart, liver, spleen, lung, and kidney in mice by HE staining (scale bar ​= ​100 ​μm).

All procedures followed the designed protocol and all mice survived well (Fig. 1a). The macroscopic of tibia plateaus were displayed in Fig. 1b while a much rougher surface with fibrillation was detected in DMM mice. According to the macroscopic of tibia plateaus in the PTH (1–34)-treated mice, the surface of tibia plateaus seems with less wear or tear, which implied with less lesion of the articular cartilage. And therefore, the wear and tear were alleviated in the PTH (1–34)-treated mice (Fig. 1c). In addition, the macroscopic scoring implied that cartilage wear was alleviated by PTH (1–34) at the 12th week. The biosafety of PTH (1–34) is favorable because no significant differences were observed in major organs in all groups according to the historical evaluations (Fig. 2).

### 3.2 The cartilage-protective effect of PTH (1–34)

**Figure 3:**
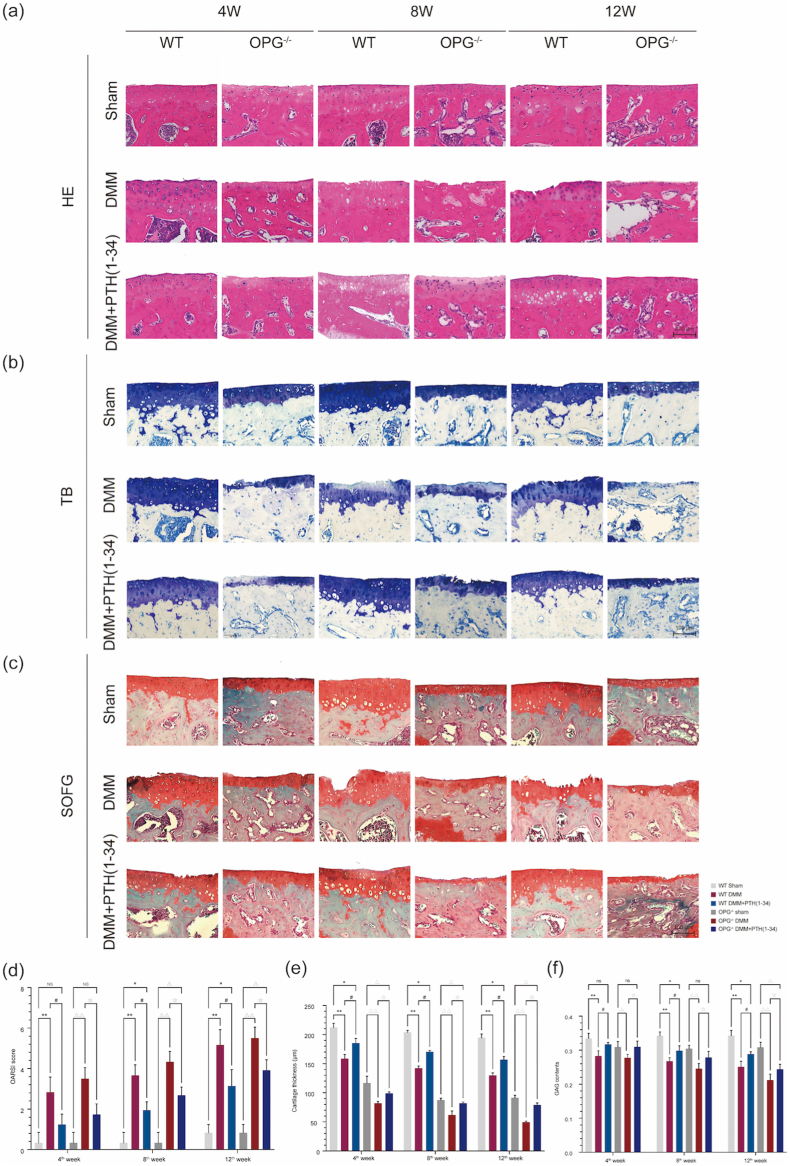
PTH (1–34) attenuates the KOA progression by restoring cartilage thickness and retaining GAG content. Representative photographs for (a) HE staining, (b) TB staining, and (c) SOFG staining (scale bar ​= ​100 ​μm). (d) OARSI score, (e) cartilage thickness, and (f) GAG contents were displayed as well. Each column represents the mean ​± ​SD (n ​= ​6). ∗, *P* ​< ​0.05; ∗∗, *P* ​< ​0.01 compared with the WT-sham group. #, *P* ​< ​0.05; ##, *P* ​< ​0.01 compared with the WT-DMM group. △, *P* ​< ​0.05; △△, *P* ​< ​0.01 compared with the OPG^−/−^-sham group. ☆, *P* ​< ​0.05; ☆☆, *P* ​< ​0.01 compared with the OPG^−/−^-DMM group.

**Figure 4:**
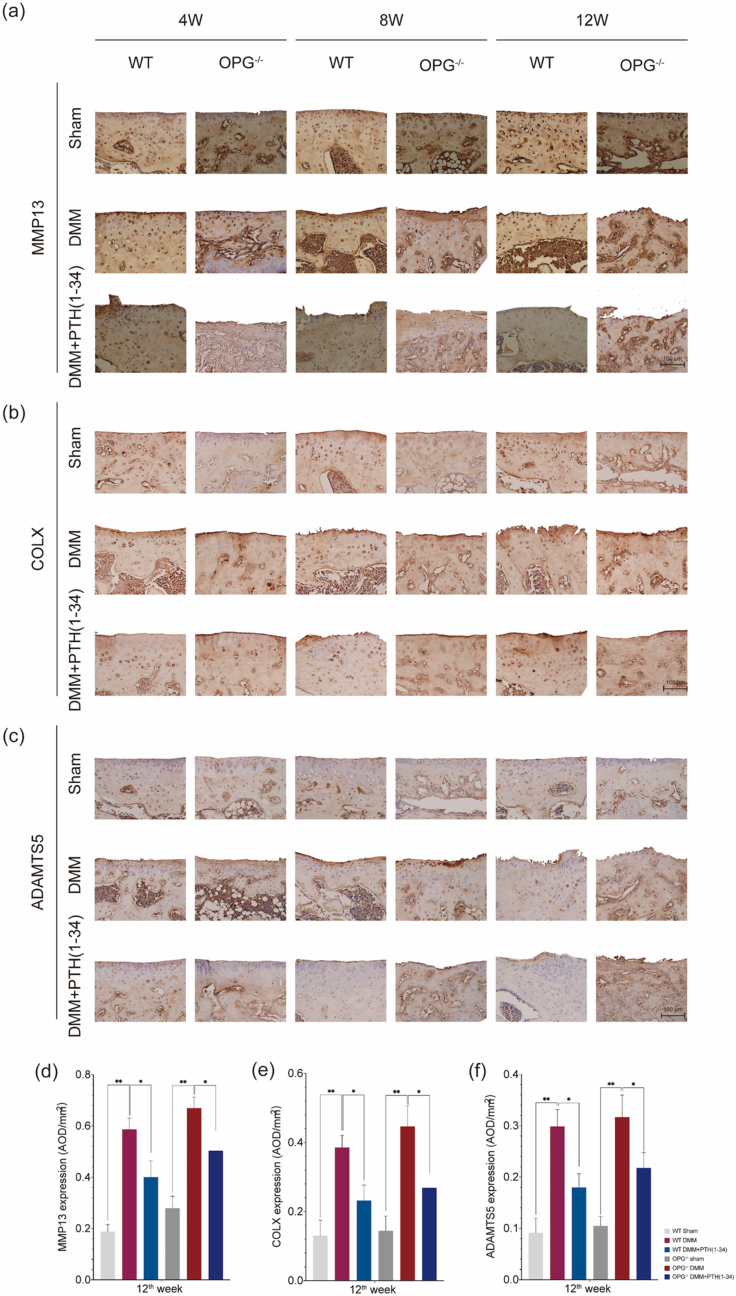
Expression of MMP13, COLX, and ADAMTS5 from the study joints. The expression of (a) MMP13, (b) COLX, and (c) ADAMTS5 could be detected in cartilage. The DMM mice with higher expression but the DMM ​+ ​PTH (1–34) mice with lower expression, which implied that PTH (1–34) could alleviate the cartilage damage in both WT and OPG^−/−^ mice. (d) to (f) The relative expression of MMP13, COLX, and ADAMTS5. Each column represents the mean ​± ​SD (n ​= ​6). ∗, *P* ​< ​0.05, ∗∗, *P* ​< ​0.01, significant difference compared with the sham group.

No degeneration of cartilage with similar smooth surface was demonstrated in sham mice while the worst situations were detected in DMM mice with cartilage surface irregularities, rough, crack, strip, or even defect according to the HE staining, the TB staining, and the SOFG staining (Fig. 3a–c). Particularly, *thinner cartilage* with fewer GAG contents were detected in *OPG−/− mice and more aggressive KOA progression* the *OPG*−/−-*DMM mice*. All DMM mice exhibited cartilage damage, but they were alleviated after the administration of PTH (1–34) differently in WT and OPG^−/−^ *mice*. The OARSI score ranked highest in all the DMM mice but reduced significantly after the treatment (Fig. 3d). In addition, the thickness of cartilage in DMM mice was increased after the administration of PTH (1–34) as well (Fig. 3e). Similarly, significant loss of GAG contents was observed in DMM mice but increased after the treatment of PTH (1–34) (Fig. 3f). Moreover, the staining of Alican Blue and Masson implied similar results (Fig. S3). We further detected the expression of MMP13, COLX, and ADAMTS5 which are key factors for affecting the development of OA. The upregulated expression of MMP13, COLX, and ADAMTS5 were noticed in the DMM mice but they were downregulated after the treatment by PTH (1–34). Moreover, the expression of MMP13, COLX, and ADAMTS5 from the IHC study implied that the OA progression in both WT and OPG^−/−^ mice could be delayed by PTH (1–34) (Fig. 4). Above all, the pathological change exhibited reducing OARSI score, restoring cartilage thickness, and increasing GAG contents after the administration of PTH (1–34) in both WT and OPG^−/−^ DMM mice.

### 3.3 PTH (1–34) ameliorates the deterioration of the SCB

**Figure 5:**
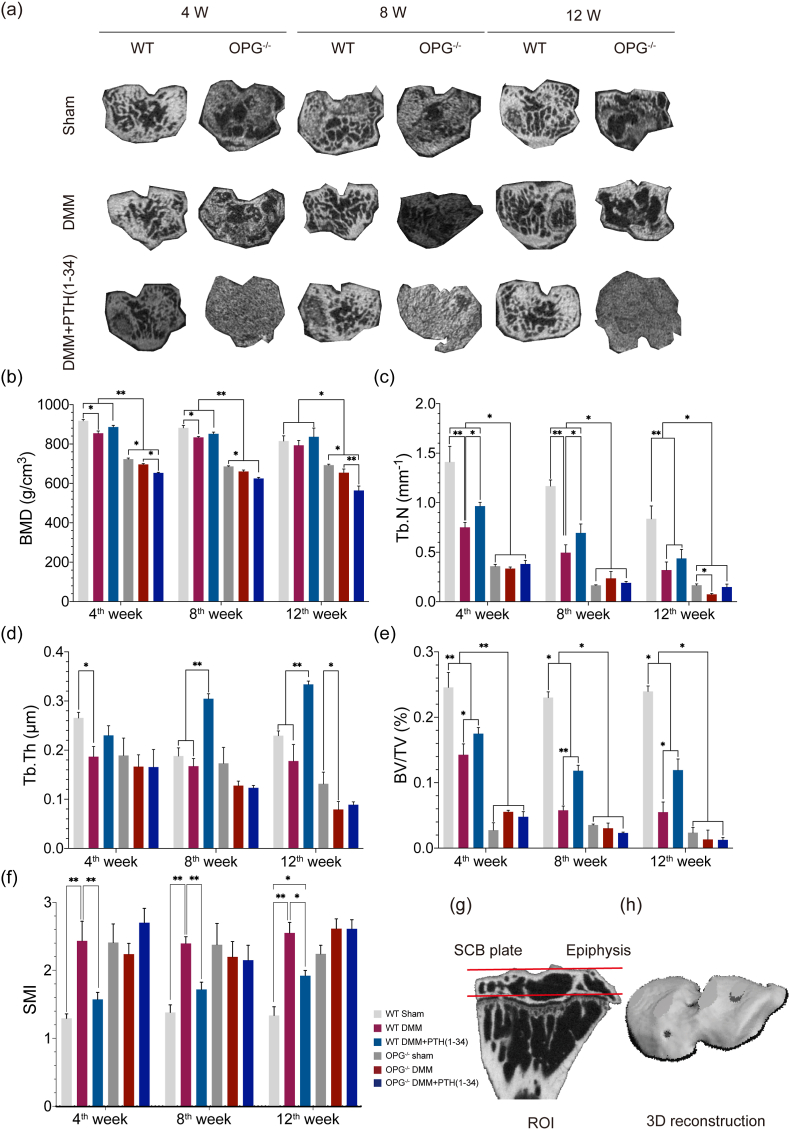
Images and evaluation of the SCB from Micro-CT. (a) Representative images of the SCB from Micro-CT. Comparison of the bone parameters from SCB including (b) BMD, (c) Tb.N, (d) Tb.Th, (e) BV/TV, and (f) SMI. (g) Illustration of the ROI in SCB. (h) 3D reconstruction of ROI in SCB.

Representative images of Micro-CT were displayed in Fig. 5a. Both WT-DMM and OPG^−/−^-DMM mice had significantly lower BMD, Tb.N, Tb.Th, and BV/TV but higher SMI at the 8th and the 12th weeks (Fig. 5b–f). The deteriorated microarchitecture was inversed and abnormal remodeling of the SCB was inhibited only in the WT-DMM ​+ ​PTH (1–34) mice rather than OPG^−/−^-DMM ​+ ​PTH (1–34) mice. Moreover, the microarchitecture of SCB shows little response to the treatment of PTH (1–34) in the OPG^−/−^-DMM ​+ ​PTH (1–34) mice. Schematic illustrations of the ROI and 3D reconstruction were displayed (Fig. 5g and h). Above all, the remarkable anabolic effects of PTH (1–34) on SCB might depend on OPG.

### 3.4 The effects of PTH (1–34) are associated with the PTH1R/OPG/RANKL/RANK signaling

**Figure 6:**
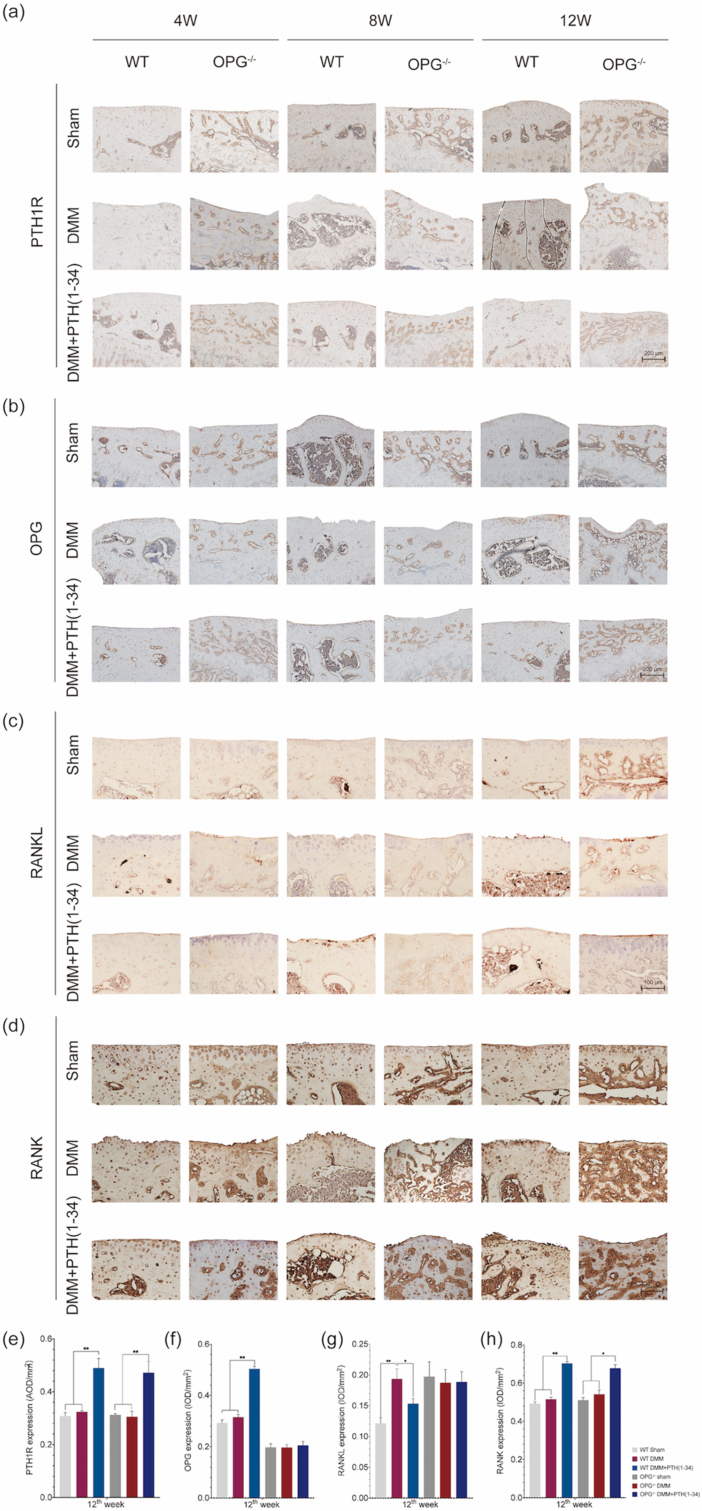
Expression of PTH1R and OPG/RANKL/RANK from the study joints. (a) The expression of PTH1R could be observed in both cartilage and SCB in all mice and it was up-regulated after the PTH (1–34) treatment. (b) The expression of OPG could only be detected in WT mice and it was up-regulated after the PTH (1–34) treatment. (c) The expression of RANKL could be detected in all mice and it was down-regulated after the PTH (1–34) treatment. (d) The expression of RANK could be observed in both cartilage and SCB in all the mice and it was up-regulated after the PTH (1–34) treatment. (e) to (h) The relative expression of PTH1R, OPG, RANKL, and RANK. Each column represents the mean ​± ​SD (n ​= ​6). ∗, *P* ​< ​0.05, ∗∗, *P* ​< ​0.01, significant difference compared with the sham group.

According to the IHC study in Fig. 6, the expression of PTH1R and RANK were up-regulated in both WT-DMM ​+ ​PTH (1–34) mice and OPG^−/−^-DMM ​+ ​PTH (1–34) mice significantly (Fig. 6a and e and Fig. 6d and h). Compared with the OPG^−/−^ mice, the expression of OPG was only upregulated in the WT-DMM ​+ ​PTH (1–34) mice. Compared with the WT-DMM mice, the expression of RANKL was downregulated remarkably. Particularly, no significant differences of the OPG and RANKL were detected in the OPG^−/−^ mice and they responded little to the treatment of PTH (1–34) in the OPG^−/−^-DMM ​+ ​PTH (1–34) mice (Fig. 6b and f, Fig. 6c and g). The IHC study implied that the therapeutic effect of PTH (1–34) was associated with the expression of PTH1R/OPG/RANKL/RANK signaling.

### 3.5 The proliferation and the synthesis of ECM were promoted by PTH (1–34) *in vitro*

**Figure 7:**
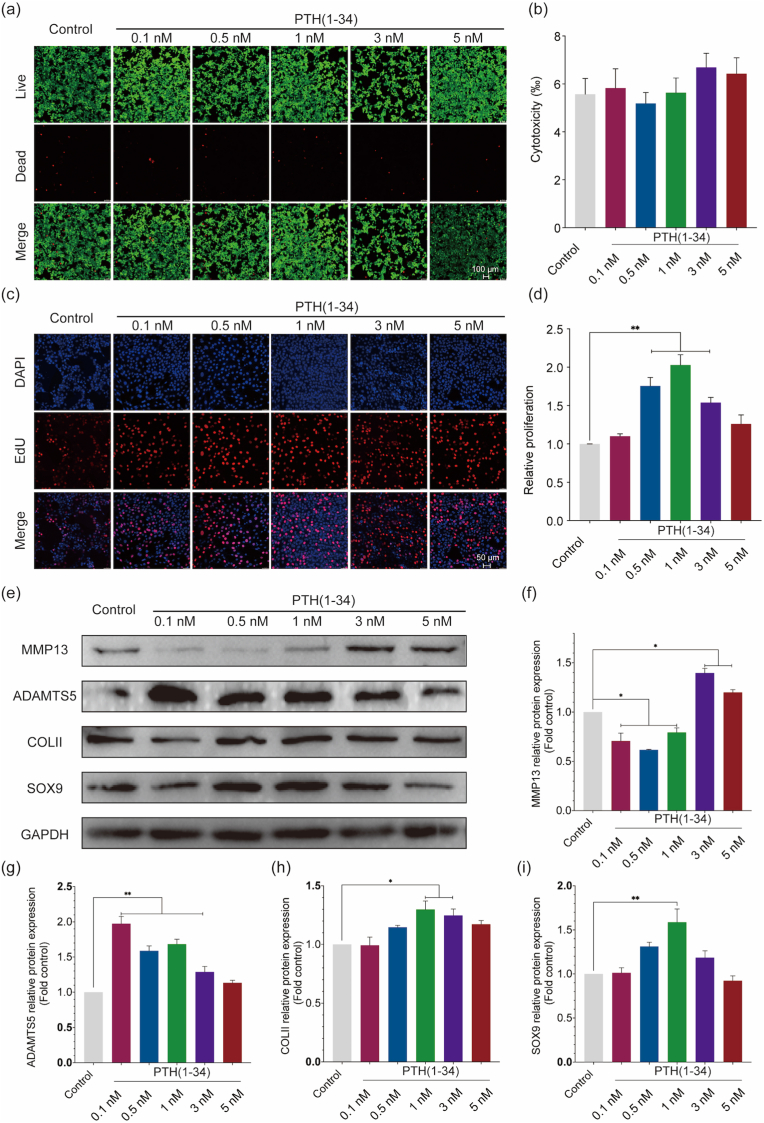
**PTH (1–34) promotes the proliferation and enhances the ECM synthesis in ATDC5 chondrocytes.** (a) and (b) representative fluorescence images from Live/Dead staining assay (scale bar ​= ​100 ​μm) and quantitative of dead cells. (c) and (d) representative photographs from EdU staining assay (scale bar ​= ​50 ​μm) and quantitative of neoplastic cells. (e) to (i) Representative proteins expression and their comparison (MMP13, ADAMTS5, COLII, and SOX9). Each column represents the mean ​± ​SD from 3 repeated and independent experiments. ∗, *P* ​< ​0.05, ∗∗, *P* ​< ​0.01.

Similar dead cells (red dots) were observed according to the Live/Dead staining (Fig. 7a and b), which showed no significant cytotoxicity in the current concentrations. Neoplastic cells labeled with the EdU (red) implied that the PTH (1–34) at 0.5 ​nM (130%), 1 ​nM (190%), and 3 ​nM (150%) would promote the proliferation (Fig. 7c and d). The MMP13 expression was down-regulated while COLII and SOX9 expression were up-regulated at protein level at the concentration of 1 ​nM (Fig. 7e–i). What's more, the expression of proteins including OPG/RANKL/RANK were checked. We found that only the RANK was expressed (Fig. S4) but no significant difference was detected after the stimulation of PTH (1–34). Above all, PTH (1–34) could increase the proliferation and enhance the ECM anabolism in the ATDC5 chondrocytes. The *in vitro* study implied that the chondro-protective effect of PTH (1–34) might be independent of OPG, which is in consistent with the *in vivo* study.

### 3.6 The catabolism of ECM and secretion of inflammatory cytokines were inhibited by PTH (1–34) *in vitro*

**Figure 8:**
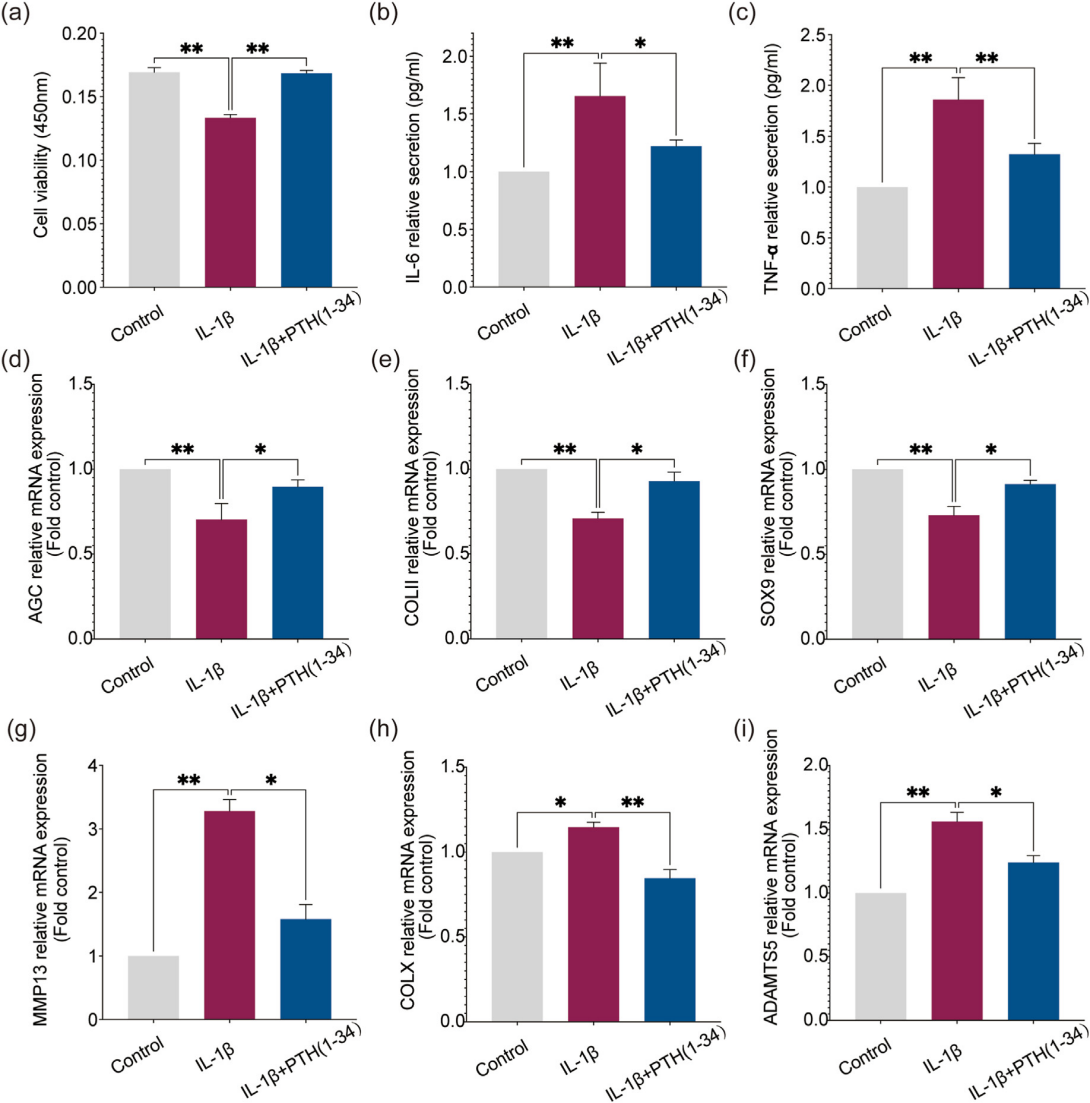
**PTH (1–34) inhibits inflammatory cytokines secretion but enhances ECM synthesis in IL-1β-induced-ATDC5 chondrocytes.** (a) Cell viability was assessed by CCK8. (b) and (c) TNF-α and IL-6 evaluated by ELISA. (d) to (i) relative mRNA expression from RT-qPCR (AGC, COLII, SOX9, MMP13, COLX, and ADAMTS5). Each column represents Each column represents the mean ​± ​SD from 3 repeated and independent experiments. ∗, *P* ​< ​0.05, ∗∗, *P* ​< ​0.01.

The viability of chondrocytes induced by IL-1β (10 ​ng/ml) might be due to the increased apoptosis but then it was increased after PTH (1–34) treated (Fig. 8a). Both the secretion of IL-6 and TNF-α were found to up-regulated in the IL-1β-induced-ATDC5 chondrocytes but they were down-regulated after the PTH (1–34) treated (Fig. 8b and c). The anabolic mRNA expression (AGC, COLII, and SOX9) would be up-regulated (Fig. 8d–f) while the ECM catabolism (MMP13, COLX, and ADAMTS5) decreased after the stimulation of PTH (1–34) (Fig. 8g–i). The adverse effect caused by IL-1β were significantly inversed after treatment by PTH (1–34).

## 4 Discussion

In the present study, the administration of PTH (1–34) exhibits a potential effect on preventing KOA progression by ameliorating the degeneration and wear of cartilage, restoring the thickness of cartilage, retaining the content of GAG, and affecting the expression of PTH1R/OPG/RANKL/RANK signaling *in vivo*. What's more, PTH (1–34) exhibits a chondro-regenerative effect by promoting viability, decreasing the secretion of inflammatory cytokines, and enhancing the ECM anabolism in chondrocytes *in vitro*. PTH (1–34) attuned KOA progression by alleviating the cartilage degeneration and SCB deterioration effectively, which suggested PTH (1–34) might be a promising therapy for KOA.

KOA is a progressive disease but the currently available treatments fail to alleviate the deterioration effectively [22]. Poor muscle function and physical performance are strong predictors relevant to symptoms clinically while the control of risk factors is essential for delaying the progression of KOA [23]. Strategies to develop disease-modifying-treatments and newly established KOA medications have attracted much attention, which would reduce the costs and benefit patients. PTH (1–34) was used to treat OP, improve healing of the bone fracture widely [24], and emerge as a promising agent for KOA treatment. Therefore, PTH (1–34) might be a medication for KOA but the potential mechanism of its pharmacological effects remains to be explored.

PTH (1–34) was demonstrated to be effective in preventing the degeneration of cartilage. The wear of the cartilage in all the DMM mice was ameliorated after the treatment of PTH (1–34). Furthermore, the reduced cartilage thickness and the loss of GAG contents were observed in the DMM mice in the 4th, 8th, and 12th weeks, which implied the progression of KOA evolved with time. The chondro-regenerative effect after the administration of PTH (1–34) is correlated with the regeneration, an increase in the cartilage thickness, and enhancement of the GAG. The articular cartilage lacks intrinsic repair capacity, moreover, the restoration of damaged tissue was hard to duplicate its original structure and composition [25]. PTH (1–34) exhibits cartilage protection and chondrocyte regeneration, which was in line with a previous study [26]. Possible mechanisms might be the inhibition of hypertrophic differentiation or the reduction of apoptosis in chondrocytes [27]. The HE staining from primary organs and no significant difference was found, which was neither specific nor sensitive compared with blood chemistry examination but supported the biosafety of PTH (1–34) in mice [28]. No malignancy pathology was observed varied from the previous report, which might be possible due to the species-specific [29]. OPG is a member of the TNF receptor superfamily in maintaining the SCB and articular cartilage. Intra-articular injection of PTH (1–34) alleviated KOA by directly protecting cartilage [30] rather than affecting the SCB [31]. Liu et al. proved that OPG played an important role in maintaining homeostasis of articular cartilage in the femoral head [32]. The deficiency of the OPG caused thinner cartilage and extensive remodeling of the SCB, which was approved in our current study. The supplementation of the PTH (1–34) improved the thickness and GAG of cartilage as well as the enhanced synthesis of ECM in both WT and OPG^−/−^ mice, which implied that the therapeutic roles of PTH (1–34) might depend on OPG.

PTH (1–34) exhibited a significantly protective effect on SCB. KOA is characterized by cartilage degeneration while the deterioration and abnormal remodeling of SCB affect the KOA progression as well [33]. The micro-architecture of the SCB was clear and distinguishable in the WT mice, which was noticed to be markedly different in the OPG^−/−^ mice. PTH (1–34) benefited the OPG^−/−^ mice in preventing the degeneration of cartilage rather than the alleviation of SCB, which implied that PTH (1–34) might be associated with the PTH1R/OPG/RANK/RANKL signaling. Moreover, the therapeutic eccect of PTH (1–34) in ameliorating the deterioration of SCB might be independent on OPG.

The OPG/RANKL/RANK pathway was identified as a critical regulator of SCB remodeling and relevant gene deficiencies, or mutations will result in abnormal bone metabolism [34]. Remodeling of osteoclasts and osteoblasts was repeated continuously in bone. Osteoblasts mediate OPG to prevent the resorption of bone while the deficiency of OPG generates the onset of bone loss or even OP [35]. The historical evaluation indicated that damage of the cartilage degenerated over time, which was in line with that the deterioration of SCB being closely associated with OA progression [36]. Moreover, the deterioration of the SCB would be a risk factor for KOA progression but the amelioration of the SCB might provide a valuable basis for the treatment of KOA [5]. The OPG^−/−^ mice with serious abnormal situations of the bone and the SCB had no response to the treatment of PTH (1–34). According to our investigation, PTH (1–34) might be dependent on OPG to alleviate the deterioration of SCB and inhibit the KOA progression. Thus, pathological change in SCB might serve as a mechanism in the OPG^−/−^ mice. Moreover, a conditional knockout approach would be very useful to dissect tissue speciﬁc roles of OPG in bone and cartilage.

Consistent with the *in vivo* study, strategies to inhibit hypertrophic maturation of chondrocytes and enhance the synthesis of ECM represent potential new therapeutic modalities, an excellent chondro-protective effect had been approved *in vitro* studies. The chondrocytes are responsible for the maintenance of the articular cartilage [37], which would provide a valid strategy for the prevention and treatment of KOA [38]. Previous investigations implied that PTH (1–34) could enhance the proliferation of chondrocytes [39], which was replenished in our current study. PTH (1–34) increased proliferation and enhance ECM anabolism of chondrocytes by upregulating COLII and SOX9 but downregulating MMP13 at the protein level. Degeneration of the chondrocytes was observed after the stimulation with IL-1β, which might be associated with oxidative stress and apoptosis. Proinflammatory parameters are molecular characteristics of KOA and are responsible for ECM catabolism and cartilage degradation [40]. In particular, the anabolic genes are essential for the integrity of the ECM while the catabolic genes are involved in cartilage degeneration, which was consistent with the relevant protein expression. All adverse effects of chondrocytes caused by IL-1β were reversed after the stimulation by PTH (1–34), which possessed the desirable chondro-protective potential for inflammatory-induced chondrocytes degeneration. Moreover, the expression of OPG/RANKL/RANK were checked via WB and only the RANK expression was detected with no significant differences. The *in vitro* studies implied that the chondro-protective of PTH (1–34) might be independent on the OPG, which was in consist with the *in vitro* study.

## 5 Conclusion

In summary, we conclude that the PTH (1–34) effectively prevents KOA progression by alleviating degeneration of cartilage and ameliorating abnormal remodeling of SCB. The cartilage and SCB were alleviated in the WT mice, but the effect of cartilage-protection observed well in the OPG^−/−^ mice. This implies that the PTH (1–34) may play excellent therapeutic role depends on the OPG in the SCB but not depend on the OPG in the cartilage. Moreover, the chondro-regenerative and chondro-protective characteristic features were well confirmed *in vitro*. The current study provides evidence that the PTH (1–34) might be a promising and potential medication for KOA.

## Funding

This study was supported by grants from 10.13039/501100001809National Natural Science Foundation of China (No. 82172432), Guangdong Basic and Applied Basic Research Foundation (No. 2021A1515012586), 10.13039/501100004791Shenzhen Key Medical Subject (No. SZXK023), Shenzhen “San-Ming” Project of Medicine (No. SZSM201612092).

## Credit authors statement

Guoqing Li: Investigation, Methodology, Data curation, Formal analysis, Writing – original draft, Writing-review, Editing. Su Liu: Investigation, Methodology, Writing-review, Editing. Yixiao Chen: Investigation, Methodology. Huihui Xu: Methodology, Writing-review, Editing. Tiantian Qi: Methodology. Ao Xiong: Investigation. Deli Wang: Investigation. Fei Yu: Investigation, Resources, Review. Jian Weng: Investigation, Resources, Review. Hui Zeng: Investigation, Conceptualization, Supervision, Funding acquisition, Resources, Review, Editing.

## Ethics approval

All surgical procedures were performed following the instructions approved by the Institutional Animal Care and Use Committee of Peking University Shenzhen Hospital (No. 2021–501).

## Availability of data and materials

Data are available from the corresponding authors upon reasonable request with the permission of Department of bone and joint in Peking University Shenzhen Hospital.

## Consent for publication

The manuscript has not been previously published and is not being concurrently submitted elsewhere.

## Declaration of competing interest

The authors declare that they have no conflict of interest.
