# Corrigendum to ‘Teriparatide ameliorates articular cartilage degradation and aberrant subchondral bone remodeling in DMM mice’ [J. Orthop. Transl.] **38** (2023) 241–255]

**DOI:** 10.1016/j.jot.2023.04.001

**Published:** 2023-04-27

**Authors:** Guoqing Li, Su Liu, Yixiao Chen, Huihui Xu, Tiantian Qi, Ao Xiong, Deli Wang, Fei Yu, Jian Weng, Hui Zeng

**Affiliations:** a Department of Bone & Joint Surgery, Peking University Shenzhen Hospital, Shenzhen, 518036, PR China; b National & Local Joint Engineering Research Center of Orthopaedic Biomaterials, Peking University Shenzhen Hospital, Shenzhen, 518036, PR China

The authors regret “an error occurred in the ***Fig. S3**. PTH (1-34) attunes the KOA progression by restoring cartilage thickness and retaining GAG content from (a) Alican Blue staining and (b) Masson staining*.” in *Appendix A. Supplementary data* in our published paper. We found an error of DMM OPG^-/-^ mice at the 8^th^ week and the 12th week in Alican Blue staining. We now have corrected the **Fig. S3** and used the right histology evaluation (DMM OPG^-/-^ mice at the 12th week). Of note that, the correction of the **Fig. S3** would have no influence on the conclusion of the current study.

The authors would like to apologise for any inconvenience caused.

Here is the original published one (**Fig. 1**) for *Appendix A. Supplementary data Fig. S3. An error occurred in the DMM OPG*^*-/-*^ *mice at the 8th week and the 12th week.*

**Figure:**
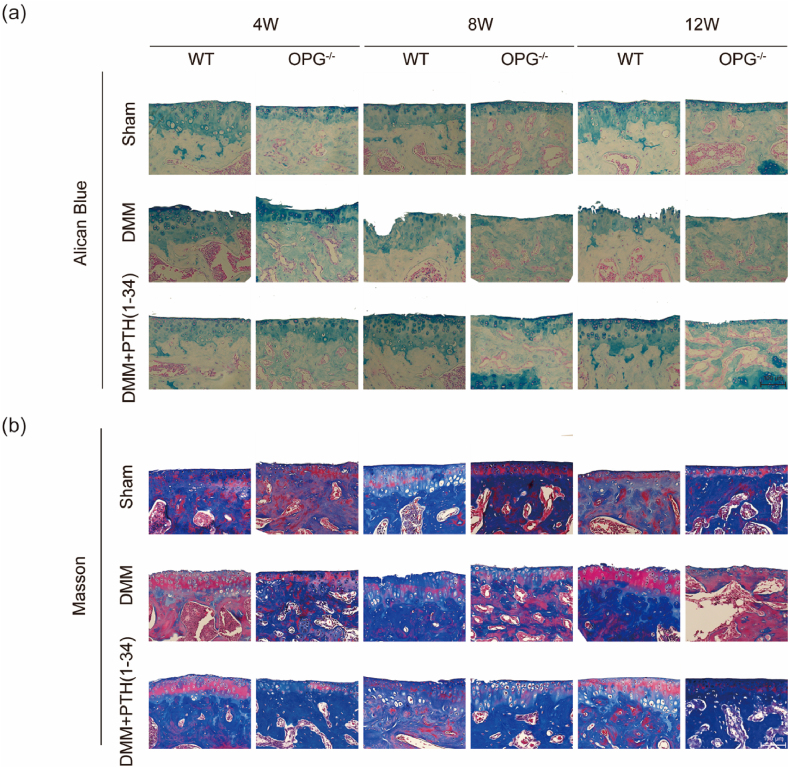
**Fig. 1** The original published one for Appendix A. Supplementary data Fig. S3.

**Figure:**
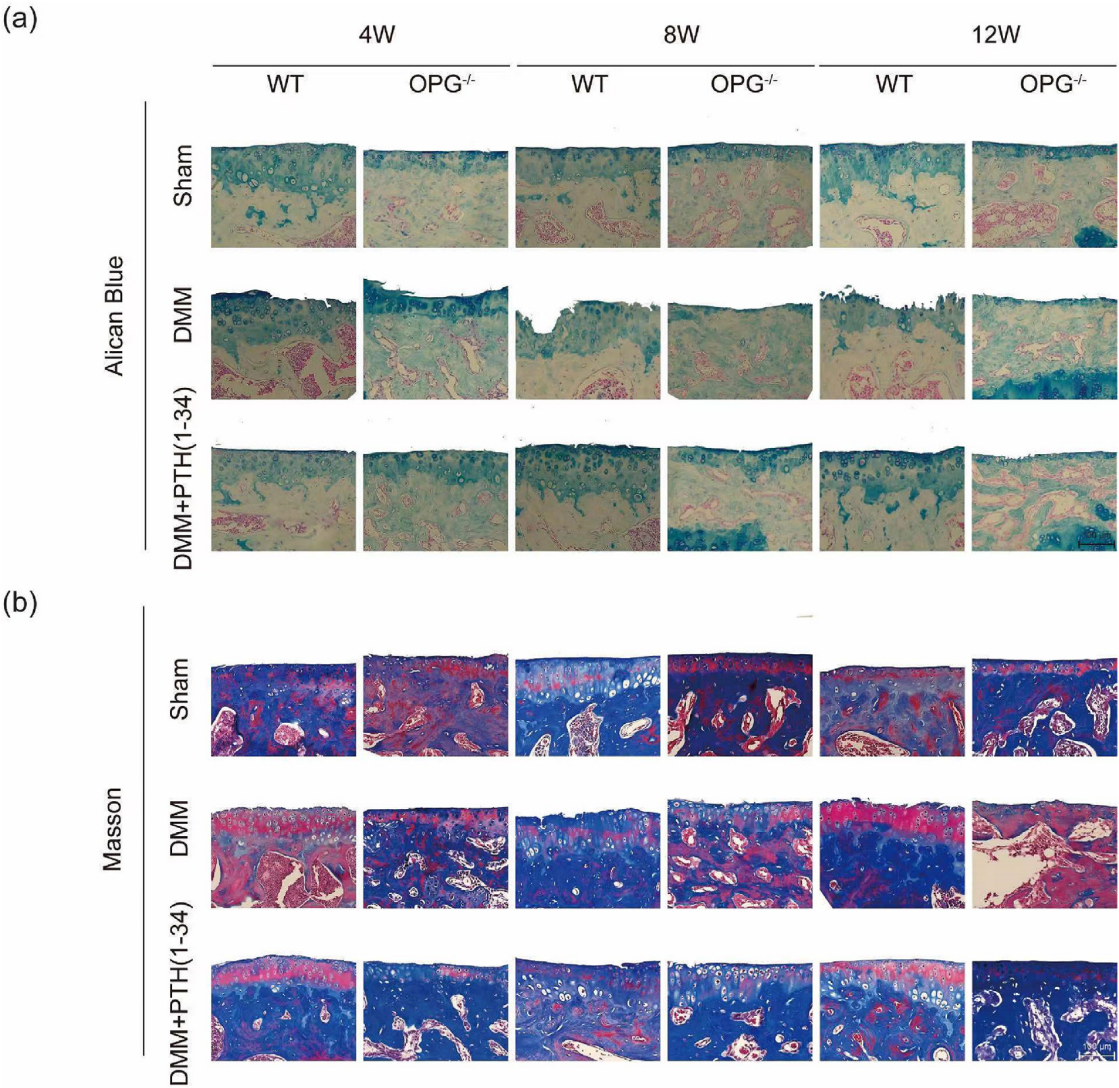
**Fig. 2** The amended figure for Appendix A. Supplementary data Fig. S3.

Here is the amended figure (**Fig. 2**) for *Appendix A. Supplementary data Fig. S3. We used the corrected histology evaluation (DMM OPG*^*-/-*^ *mice at the 12*^*th*^ *week) in Alican Blue staining*.

