# Supplementary material for: Teriparatide ameliorates articular cartilage degradation and aberrant subchondral bone remodeling in DMM mice
Source: J Orthop Translat. 2022 Dec 7;38:241–55. doi: 10.1016/j.jot.2022.10.015 (PMC9731868; doi:10.1016/j.jot.2022.10.015)
Supplement: Multimedia component 1 [file mmc1.docx]

| **Table S1**  Macroscopic grading system | |
| --- | --- |
| Grade | Contents |
| 0 | Normal |
| 1 | Surface roughening |
| 2 | Fibrillation and fissures |
| 3 | Large erosions extending down to the subchondral bone |
| 4 | Larger erosions down to the subchondral bone |
