# Supplementary material for: Teriparatide ameliorates articular cartilage degradation and aberrant subchondral bone remodeling in DMM mice
Source: J Orthop Translat. 2022 Dec 7;38:241–55. doi: 10.1016/j.jot.2022.10.015 (PMC9731868; doi:10.1016/j.jot.2022.10.015)
Supplement: Multimedia component 2 [file mmc2.docx]

| **Table S2**  The recommended semi-quantitative scoring system | |
| --- | --- |
| Grade | Osteoarthritic damage |
| 0 | Normal |
| 0.5 | Loss of Safranin-O without structural changes |
| 1 | (Superficial) Small ﬁbrillations without loss of cartilage |
| 2 | Vertical clefts down to the layer immediately below the superﬁcial layer and some loss of surface lamina |
| 3 | (Mild) Verical clefts/erosion to the calciﬁed cartilage extending to <25% of the articular surface |
| 4 | (Moderate) Vertical clefts/erosion to the calciﬁed cartilage extending to 25%～50% of the articular surface |
| 5 | (Severe) Vertical clefts/erosion to the calciﬁed cartilage extending to 50%～75% of the articular surface |
| 6 | (Eburnation) Vertical clefts/erosion to the calciﬁed cartilage extending >75% of the articular surface |
